# Epidemiology of Supernumerary Teeth in 5000 Radiography Films: Investigation of Patients Referring to the Clinics of Ardabil in 2015–2020

**DOI:** 10.1155/2021/6669436

**Published:** 2021-02-22

**Authors:** Emran Hajmohammadi, Samira Najirad, Hesam Mikaeili, Aziz Kamran

**Affiliations:** ^1^Department of Oral and Maxillofacial Surgery, School of Dentistry, Ardabil University of Medical Sciences, Ardabil, Iran; ^2^School of Medicine and Allied Medical Sciences, Ardabil University of Medical Sciences, Ardabil, Iran

## Abstract

**Background:**

Supernumerary tooth is defined as any extra tooth or odontogenic structure that is formed on normal dentition. Supernumerary teeth cause such problems as deficiency in tooth growth, ectopic growth, displacement, crowding, diastema, odontogenic cyst formation, decay of the adjacent tooth, malocclusion, and esthetic problems. This study was conducted aiming at determining epidemiology of supernumerary teeth in Ardabil city in 2020. *Materials & Methods*. In this retrospective descriptive analytical study, 5000 panoramic radiographs of patients referring to Rad and Baser Oral and Maxillofacial Radiology Centers were selected through multistage sampling method during 2015–2020. The data were collected by checklist and analyzed with using SPSS-21 and chi-squared, Fisher, and one-way ANOVA tests with a significance level less than 5%.

**Results:**

The prevalence of supernumerary teeth was estimated as 1.06% (*n* = 53), and no significant difference between the males and females was seen. Most supernumerary teeth were found in the distomolar (44.1%) and parapremolar (29.4%) locations. The majority of supernumerary teeth were present in the maxilla (73.5%) and were impacted (77.9%) and unilateral (71.7%). The number of supernumerary teeth was 68 cases and majority of patients (86.8%) had one supernumerary tooth.

**Conclusion:**

Supernumerary tooth in this study had a high prevalence compared to similar studies, and unlike most previous studies, the most common type of supernumerary tooth was distomolar. Early diagnosis and proper medical planning are essential for managing supernumerary teeth.

## 1. Introduction

Supernumerary tooth is defined as any extra tooth or odontogenic structure that is formed on normal dentition. This position is also called hyperdontia. Supernumerary teeth can be unilateral or bilateral, single or multiple, in any parts of the dental arch. These teeth also form in both the deciduous and permanent teeth systems [1–3].

The etiology of supernumerary teeth is not clearly defined and various theories have been reported for it, the most common theory being the formation of teeth as a result of horizontal growth and hyperactive dental lamina [1, 4, 5]. In general, a combination of environmental and genetic, factors has been proposed to explain the occurrence of supernumerary teeth [6]. The prevalence of supernumerary teeth in different populations in permanent dentition is between 0.5% and 5.3% and in deciduous teeth is between 0.2% and 0.8% [4, 7, 8]. In general, the prevalence of supernumerary tooth is higher in the Asian population [9–11]. The prevalence of hyperdontia in countries between the Caspian Sea and the Black Sea is about 0.1% to 3.8% and has a higher prevalence (between 0.4% and 3.8%) in the Arab and East Asian countries [12–14].

Supernumerary teeth can cause problems such as defects in tooth growth, ectopic growth, displacement, crowding, diastema, odontogenic cyst formation, decay of the neighboring tooth, malocclusion, and esthetic problems [15]. Supernumerary teeth may be associated with a variety of syndromes, including Gardner syndrome, EhlersDanlos syndrome, Cleidocranial dysplasia, and AndersonFabry [1, 6]. Supernumerary teeth often manifest in many forms. However, these teeth may also develop in patients without the syndrome. These teeth may present as single teeth, two teeth, or multiple teeth, or as unilateral or bilateral [1, 9]. Also, the presence of an undeveloped supernumerary tooth can make the site unsuitable for possible future implants and make implant placement difficult [16].

Since the majority of supernumerary teeth (93–80%) can cause clinical complications, early detection and orthodontic and surgical interventions are of significant value in reducing future clinical problems and establishing proper occlusion of adjacent permanent teeth [12, 15].

Familiarity with these teeth is important for orthodontists, pediatricians, and general dentists, who usually see children at an early age and can be more effective for early diagnosis and planning for long-term multifaceted treatment [4].

Knowing the prevalence and pattern of supernumerary teeth can be helpful in the timely diagnosis and prevention of dental malformations side effects by dentists. Therefore, this study was presented to investigate the frequency of supernumerary tooth in Ardabil.

## 2. Materials and Methods

This descriptive cross-sectional study was performed on the 5000 panoramic radiographs from the archives of two specialized centers of maxillofacial radiology, i.e., Rad and Baser from 2015 to 2020 in Ardabil. In total, 36,340 panoramic radiographs were recorded in two centers (26,440 radiographs in the center of Baser and 9900 radiographs in the center of Rad). The multistage sampling method was used for sampling. In the first stage, according to the total number of radiographs in these two centers, the number of samples in each center was calculated and allocated as a quota.

In the second stage, the regular random sampling method was used to select the samples, so that by presenting the number from the extracted list, a number to the radiographs were assigned and one radiograph out of every 10 numbers were regularly selected. To extract the data, a checklist prepared based on specific objectives was used. Checklists included variables of gender/type of jaw/unilateral, bilateral/type of growth/location/single, double, multiple/number of supernumerary teeth. Thus, by observing the selected radiographs in the sampling method, if there was a supernumerary tooth, the checklist was completed.

Descriptive and inferential statistics were used to analyze the data. To report the number and percentage of supernumerary teeth, relative frequency, mean, and standard deviation were used, and to compare the status of supernumerary teeth based on unilateral and bilateral, type of growth (erupted, impacted), location by sex, and type of jaw, chi-squared and Fisher tests were used. To compare the number of supernumerary teeth by type of jaw, sex, and type of growth, the independent *t* test was used. To compare the number of supernumerary teeth by single, double, multiple and unilateral and bilateral, midline, one-way analysis of variance was used. The tests were analyzed with a significance level of less than 5% using SPSS software version 21.

## 3. Results

The results showed that out of the total of 5000 subjects, 2726 (54.5%) were female and 2274 (45.5%) were male. The prevalence of supernumerary teeth in this study was 1.06% (53 patients), and out of 53 patients with supernumerary teeth, 29 (54.7%) were female and 24 (45.3%) were male. Also, 46 patients (86.8%) had one supernumerary tooth, 4 patients (7.5%) had 2 supernumerary teeth, 1 patient (1.9%) had 3 supernumerary teeth, 1 patient (1.9%) had 5 supernumerary teeth, and 1 patient (1.9%) had 6 supernumerary teeth.

Out of 53 patients with supernumerary teeth, 39 (73.6%) had maxillary supernumerary teeth, 38 cases (71.7%) had unilateral supernumerary teeth, and 38 patients (71.7%) had impacted supernumerary teeth (Table 1).

**Figure 1:**
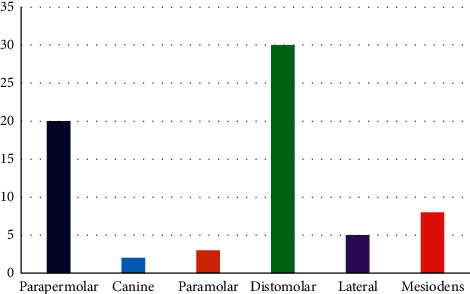
Frequency of supernumerary teeth by location.

Out of 53 patients with supernumerary teeth, 22 cases (41.5%) distomolar, 12 cases (22.6%) parapremolar, 8 cases (15.1%) mesiodens, 5 cases (9.4%) in the lateral incisor area, 2 cases (3.8%) in the canine area, 2 cases (3.8%) paramolar and 2 cases (3.8%) combined (having several types of supernumerary teeth at the same time) were seen (Figure 1).

A total of 68 supernumerary teeth were seen that 41 (60.3%) were in females and 27 (39.7%) were in males. Out of 68 supernumerary teeth, 50 teeth (73.5%) were in the maxilla and 53 teeth (77.9%) were impacted.

**Figure 2:**
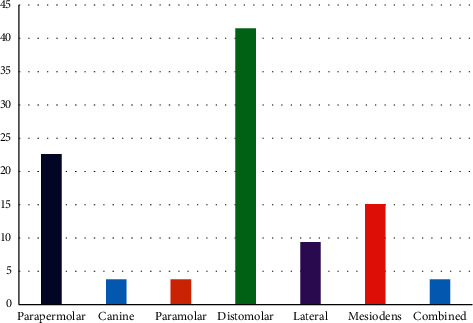
Frequency of patients with supernumerary teeth by type.

Out of the 68 supernumerary teeth, 30 (44.1%) in distomolar, 20 (29.4%) parapremolar, 8 (11.8%) mesiodens, 5 (7.4%) in the lateral incisor area, 2 (2.9%) in the canine area, and 3 cases (4.4%) of paramolar were found (Figure 2).

The number of supernumerary teeth was in average 1.28 (minimum 1 and maximum 6 supernumerary teeth) per person and the average number of supernumerary teeth in women is more than men, but this difference between the two sexes was not significant.

Table 2 shows that 82.9% (34 teeth) of supernumerary teeth in women and 59.3% (16 teeth) of supernumerary teeth in men were in the maxilla. The chi-squared test showed a significant difference in the distribution of supernumerary teeth between men and women by jaw type (*P* ≤ 0.05). Also, in both genders, the majority of supernumerary teeth were impacted, but no significant difference in this variable between the two sexes was seen (Table 3). Also, in both genders, the majority of supernumerary teeth were unilateral, and there was no significant difference in this variable between the two sexes.

The results showed that the majority of supernumerary teeth in men (45.8%) were in parapremolar location and in women (55.2%) were distomolar type, and a significant difference in terms of supernumerary teeth location was found between the males and females (*P* ≤ 0.05). (Table 4).

Also, the majority of supernumerary teeth with parapremolar location, 70% (14 teeth) were in the mandible, and 30% (6 teeth) in the maxilla, and all teeth with distomolar, mesiodens, and paramolar locations were located in the maxilla. Fisher test showed a significant difference in the location distribution of supernumerary teeth by jaw type (*P* ≤ 0.05). (Table 5).

## 4. Discussion

In this study, 5000 panoramic radiographs were examined, of which 53 patients had a total of 68 supernumerary teeth. The prevalence of supernumerary tooth was 1.06%. According to the reports presented in the literature, the frequency of supernumerary teeth in different populations was reported between 0.5% and 5.3% in permanent tooth and between 0.2% and 0.8% in deciduous teeth [1, 7, 8]. Amini et al. (2013) in Tehran reported the prevalence of supernumerary tooth as 0.72% [17], Saurabh Singh et al. (2019) in northern Malaysia reported it as 1.05% [18], Demiriz et al. (2015) in Turkey reported it as 2.14% [19], and 1.6%, 0.86%, and 1.05% in Nepal [20], Palestine [21], and Madrid [22], respectively. Variation in prevalence among populations can be attributed to racial factors, sampling method, differences in sample size, age of subjects, diagnostic tools, and selection of individuals from medical centers [23].

The present study showed that the frequency of supernumerary tooth is higher in women than in men, but this difference between the two sexes was not significant. In most studies, the frequency of supernumerary teeth in men has been reported more than women, such as the study of Singh et al. (2014), Khandelwal et al. (2018), Saurabh Singh et al. (2019), Çelikoğlu M et al. (2010), and Zahra Razavi Rouhani et al. (2018) [15, 18, 20, 24, 25].

In contrast, a number of studies such as the study of Neville et al. (2013) [13], Mansoor (2007) [16], and Amini et al. (2011) [17] did not find significant differences between men and women in this regard. But, Demiriz et al. (2017) [19] reported the prevalence of supernumerary teeth in women more than men. One of the reasons that has been stated in most studies reporting higher prevalence of supernumerary teeth in men than women can be related to the fact that X-dependent transmission has been suggested for the occurrence of hyperdontia, which can also explain the higher prevalence in men. However, a clear reason for this subject was not been found yet [17].

The present study showed that the majority of supernumerary teeth were located in the maxilla in both males and females, which is consistent with the results of few similar studies, such as those of Singh VP et al. (2014), Khandelwal et al. (2018), Saurabh Singh et al. (2019), Çelikoğlu et al. (2010), Zahra Razavi Rouhani et al. (2018), Demiriz et al. (2017).), Arandi et al. (2020), Amini et al. (2013), Vahid dastjerdi et al. (2011), Leco_Berrocal et al. (2007), and Fernández Montenegro et al. (2006) [9, 15, 17, 18, 20, 22, 24–27]. Although the reason for this was not fully understood, one reason given for it could indicate a higher prevalence of Hyperdontia in the maxillary forearm [17].

In this study, most of the supernumerary teeth were distomolar (44.1%) followed by parapremolar (29.4%) mesiodensis (11.8%), lateral incisors (7.4%), paramolar (4.4%), and canine (2.9%), respectively. This finding is in line with the study of Leco_Berrocal Mi et al. (2007), in which out of 2000 patients studied, 24 supernumerary teeth were added, of which 38% were distomolar supernumerary teeth reported as the most common type of supernumerary teeth. Our study findings are consistent with those by Leco_Berrocal [22].

This finding is inconsistent with the studies of Amini et al. (2013), Singh et al. (2014), Saurabh (2019), Çelikoğlu (2010), Arandi et al. (2020), Fernández Montenegro et al. (2006), Garvey et al. (1999), and Esenlik et al. (2007) [13, 15, 17, 18, 20, 21, 26, 28], which listed mesiodens as the most common type of supernumerary tooth. In the studies of Khandelwal et al. (2018) [24] and Luten et al. parapremolar and lateral are the most common types of supernumerary tooth, respectively [29].

It seems that the prevalence estimation of supernumerary tooth can be affected by sampling methods, genetic and environmental factors as well as the age of the subjects. According to studies, the appearance of supernumerary teeth is more common in the first 3 decades of life than in the older age groups. In studies that included mostly the younger age groups (children), the prevalence of supernumerary teeth is higher (about 1.28% to 2.4%) and mostly in the premaxillary part, but in the studies that examined the older age groups (adults), a lower prevalence (about 0.4% to 1%) was reported, which was mostly in the maxilla and posterior parts of the dental arch [30, 31].

In a study by leco_berrocal et al., the most common location of supernumerary teeth in children was in the premaxillary location and in adults was in the distomolar location [22]. On the other hand, this can be explained as follows: having supernumerary tooth in the premaxillary location, especially mesiodens, which is very effective in a person's beauty and aesthetics, it is possible that a person had extracted that tooth in childhood and therefore does not have the desired tooth in older ages. Also, one of the reasons people go to the dental office in the puberty period is wisdom tooth extraction, and it is possible that, during this time, they have noticed supernumerary distomolar teeth in that area. According to the results of the present study, distomolar supernumerary tooth is the most common among other types of supernumerary tooth. In this study, according to the explanations and results of various studies, we can guess that most of the people studied in this study were adults, and according to this case, this issue can be justified to some extent. However, the genetic and environmental factors should not be ignored.

In this study, most of the supernumerary teeth in men were in the parapermular position, and in women, most of the supernumerary teeth were in the distomular position. This finding was consistent with the study by Rajab et al. (2002), Sasaki et al. (2007), Hyun et al. (2008) [5, 32, 33]. However, in several studies, distomolar has been reported more in men, such as in studies by Kurt et al. (2015), Kaya et al. (2015), and Thomas et al. (2013), which differed from the results of the present study [34–36]. It seems that this difference can be due to racial differences and other biological and environmental factors.

In this study, 70% of parapremolar supernumerary teeth were in the mandible and 30% in the maxilla. The results also showed that all distomolar, mesiodens, and paramolar teeth were located in the maxilla. Numerous studies have reported that the incidence of distomolars in the maxilla is between 69% and 91% [37, 38]. In the study of Kaya et al. (2015), 90% of distomolars were present in the maxilla (37). Also, in the study of Kokten et al. (2003) and Menardia-Pejuan V (2000), distomolars were seen more in the maxilla than in the mandible [39, 40]. In the study of Arandi et al. (2020), all distomolars were present in maxilla [21].

Similar studies have shown that 57% to 90% of supernumerary premolars found are located in the mandible [5, 8, 19, 41]. Leco_Berrocal MI et al. (2007), Gomes et al. (2008), and Mahbob et al. (2012) mentioned the largest number of supernumerary teeth in the mandible as premolars [4, 22, 42]. In the study of Arikan et al. (2013), all premolar supernumerary teeth were present in the mandible [43].

In the present study, out of 68 extra teeth, 77.9% were impacted and 22.1% were erupted. In the study of Demiriz et al. (2015), 84% of supernumerary teeth were impacted and 16% were erupted [19]. In the study of Leco_Berrocal et al. (2007), 95.8% of supernumerary teeth were impacted and only 4.2% of them were erupted [22]. While in the study of Singh et al. (2014), 56.36% of supernumerary teeth were erupted and 43.63% of them were impacted, and in the study of Arandi et al. (2020), 52.2% were erupted and 47.8% of supernumerary teeth were impacted [20, 21].

In this study, the majority of supernumerary teeth were unilateral in both sexes. Thus, 71.7% of patients had unilateral supernumerary teeth and 13.2% had bilateral supernumerary teeth. Consistent with the results of our study, in the study of Amini et al. (2013), all supernumerary teeth were unilateral but no bilateral supernumerary teeth were seen in any of the patients [17]. In other similar studies, the majority of supernumerary teeth were unilateral [4, 5].

In this study, out of 53 patients with supernumerary teeth, 46 patients (86.8%) had single supernumerary teeth and 4 patients (7.5%) had double supernumerary teeth, and in 3 patients (5.5%), multiple supernumerary teeth were observed. According to the4 studies, single supernumerary tooth occurred in 76% to 86% of people, double supernumerary teeth occurred in 12%–23% of people, and multiple supernumerary teeth occurred in less than 1% of people [5, 10, 11]. Consistent with the results of our study, in the study of Çelikoğlu et al. (2010), out of 42 patients with supernumerary teeth, 75% (36 people) had single supernumerary tooth, 25% (6 people) had double supernumerary teeth, and no multiple supernumerary teeth was found [15]. In the study of Demiriz et al. (2015), out of 123 patients, 80.5% (*n* = 99) had single supernumerary tooth, 15.5% (*n* = 19) had double supernumerary teeth, and 0.8% (*n* = 5) had multiple supernumerary teeth [19]. In the study of Arandi et al. (2020), most patients (64.7%) had single tooth, 35.3% had two teeth, and no patient with more than two supernumerary teeth was found [21].

## 5. Conclusion

In the present study, the prevalence of supernumerary teeth in Ardabil was 1.06%, which can be said to have a high prevalence compared to similar studies. The prevalence of supernumerary teeth was not significantly different between the two sexes (*P* ≥ 0.05). The most supernumerary teeth found in this study were distomolar (44.1%), and the majority of supernumerary teeth were in maxilla (73.5%), impacted (77.9%), and unilateral (71.7%). The location of supernumerary teeth by gender in this study is inconsistent with the majority of similar studies.

### 5.1. Recommendations

Due to the high prevalence of supernumerary teeth in the study population and the consequences caused by this type of tooth, it is recommended that, in each dental care for patients requiring radiography, supernumerary tooth screening be considered to prevent subsequent problems.

### 5.2. Limitations

One of the limitations of the present study is the lack of investigation of the relationship between age and other socioeconomic variables in the prevalence of supernumerary teeth, which is due to the lack of patient information in the records. It is suggested that future studies be performed prospectively and investigate the role of these variables in estimating and predicting supernumerary teeth.

## Figures and Tables

**Table 1:** Frequency of supernumerary teeth in the study population.

Variable		*N*	Percent
Gender	Male	24	45.3
	Female	29	54.7

Jaw	Mandible	12	22.6
	Maxillary	39	73.6
	Both	2	3.8

Number	Single	46	86.8
	Double	4	7.5
	Multiple	3	5.7

Lateral status	unilateral	38	71.7
	Bilateral	7	13.2
	Midline	8	15.1

Status	Impacted	38	71.7
	Erupted	13	24.5
	Both	2	3.8

Total		53	100

**Table 2:** Comparison of the average number of supernumerary teeth by gender.

Gender	No.	Average	SD	*P* value
Female	29	1.37	1.17	0.4
Male	24	1.16	0.482	

**Table 3:** Comparison of the number of supernumerary teeth of men and women according to the type of jaw and the type of extra teeth.

Variable		Gender		Total	*P* value
		Male	Female		
Status	Impacted	21 (77.8%)	32 (78%)	41 (100%)	0.98
	Erupted	6 (22.2)	9 (22%)	27 (100%)	

Jaw (%)	Maxillary	16 (59.3%)	34 (82.9%)	18 (26.5%)	0.03
	Mandible	11 (40.7%)	7 (17.1%)	50 (73.5%)	

Total		27 (100%)	41 (100%)	68 (100%)	

**Table 4:** Comparison of type of supernumerary teeth by gender.

Gender	Type								*P*-value
	Parapremolar	Combined	Canine	Paramolar	Distomolar	Lateral	Mesiodens	Total	
Female	1 (3.4%)	1 (3.4%)	1 (3.4%)	2 (6.9%)	16 (55.2%)	3 (10.3%)	5 (17.2%)	29 (100%)	0.018
Male	11 (45.8%)	1 (4.2%)	1 (4.2%)	0	6 (25%)	2 (8.3%)	3 (12.5%)	24 (100%)	
Total	12 (22.6%)	2 (3.8%)	2 (3.8%)	2 (3.8%)	22 (41.5%)	5 (9.4%)	8 (15.1%)	53 (100%)	

**Table 5:** Comparison of the type of supernumerary teeth by jaw.

Jaw	Type							*P*-value
	Parapremolar	Canine	Paramolar	Distomolar	Lateral	Mesiodens	Total	
Mandible	14 (70%)	1 (50%)	0	0	3 (60%)	0	18 (26.5%)	*P* ≤ 0.001
Maxillary	6 (30%)	1 (50%)	3 (100%)	30 (100%)	2 (40%)	8 (100%)	50 (73.5%)	
Total	20 (100%)	2 (100%)	3 (100%)	30 (100%)	5 (100%)	8 (100%)	68 (100)	

## Data Availability

The data the questionnaire used to support the findings of this study are available from the corresponding author upon request.
